# Telephone-Based Word List Recall and Hearing Ability

**DOI:** 10.1093/geroni/igab046.2660

**Published:** 2021-12-17

**Authors:** Taylor Atkinson, Ross Andel

**Affiliations:** University of South Florida, Tampa, Florida, United States

## Abstract

Certain consonant sounds called fricatives (e.g, “s” and “f”) are difficult to hear over the telephone; phones exclude high-frequency sounds that affect their intelligibility. This may be problematic for older adults responding to phone-based memory tests. Many older adults have some degree of hearing loss, and older men have it more in the high-frequency range. Hearing loss, in combination with phone bandwidth restrictions, may reduce older adults’ recall of fricative words. Participants (n=3,612, mean age=64.2, 60% women) in the 1998 wave of the Health and Retirement study (HRS) completed a word list immediate recall task over the phone. List 4 recall was examined because it was evenly split (5 each) between words with and without fricative consonant sounds. Subjective ratings of hearing and health, age, depression, and education were also measured. A Wilcoxon signed-rank test showed participants recalled fewer fricative (M=2.8) than nonfricative (M=3.0) words, Z=-8.47, p<.001. An ordinal regression for fricative word recall indicated a sex by hearing interaction; males with worse hearing were less likely to recall more fricative words, OR=.94, 95% CI [.88, 1.01], p=.076, after controlling for age, education, health, and depression. An ordinal regression for nonfricative word recall did not show a main effect for hearing or a hearing by sex interaction. For both models, age, education, and health were related to recall. Consonant sounds may influence phone-based word recall, particularly for older men. Attention should be paid to word selection when designing phone-based cognitive tests in order to avoid memory impairment overestimation.

